# A Next-Generation Sequencing Method for Genotyping-by-Sequencing of Highly Heterozygous Autotetraploid Potato

**DOI:** 10.1371/journal.pone.0062355

**Published:** 2013-05-08

**Authors:** Jan G. A. M. L. Uitdewilligen, Anne-Marie A. Wolters, Bjorn B. D’hoop, Theo J. A. Borm, Richard G. F. Visser, Herman J. van Eck

**Affiliations:** 1 Laboratory of Plant Breeding, Wageningen University, Wageningen, The Netherlands; 2 The Graduate School for Experimental Plant Sciences, Wageningen, The Netherlands; 3 Centre for BioSystems Genomics, Wageningen, The Netherlands; University of Guelph, Canada

## Abstract

Assessment of genomic DNA sequence variation and genotype calling in autotetraploids implies the ability to distinguish among five possible alternative allele copy number states. This study demonstrates the accuracy of genotyping-by-sequencing (GBS) of a large collection of autotetraploid potato cultivars using next-generation sequencing. It is still costly to reach sufficient read depths on a genome wide scale, across the cultivated gene pool. Therefore, we enriched cultivar-specific DNA sequencing libraries using an in-solution hybridisation method (SureSelect). This complexity reduction allowed to confine our study to 807 target genes distributed across the genomes of 83 tetraploid cultivars and one reference (DM 1–3 511). Indexed sequencing libraries were paired-end sequenced in 7 pools of 12 samples using Illumina HiSeq2000. After filtering and processing the raw sequence data, 12.4 Gigabases of high-quality sequence data was obtained, which mapped to 2.1 Mb of the potato reference genome, with a median average read depth of 63× per cultivar. We detected 129,156 sequence variants and genotyped the allele copy number of each variant for every cultivar. In this cultivar panel a variant density of 1 SNP/24 bp in exons and 1 SNP/15 bp in introns was obtained. The average minor allele frequency (MAF) of a variant was 0.14. Potato germplasm displayed a large number of relatively rare variants and/or haplotypes, with 61% of the variants having a MAF below 0.05. A very high average nucleotide diversity (π = 0.0107) was observed. Nucleotide diversity varied among potato chromosomes. Several genes under selection were identified. Genotyping-by-sequencing results, with allele copy number estimates, were validated with a KASP genotyping assay. This validation showed that read depths of ∼60–80× can be used as a lower boundary for reliable assessment of allele copy number of sequence variants in autotetraploids. Genotypic data were associated with traits, and alleles strongly influencing maturity and flesh colour were identified.

## Introduction

DNA variants such as single nucleotide polymorphisms (SNPs), multinucleotide polymorphisms (MNPs), and insertions and deletions (indels) are differences at the nucleotide sequence level among individuals or alleles and represent the basic units of genetic diversity. They can be assayed and exploited as high-throughput molecular markers and are widely used for marker assisted and genomic selection, association analysis and mapping of quantitative trait loci (QTL), and haplotype and pedigree analysis in several crops and model plants [1]–[7]. Genotyping of DNA sequence variants in highly heterozygous polyploid species, such as potato (*Solanum tuberosum*), is more challenging than in diploid species, because a given gene may be represented by up to four different alleles per locus per genotype. Therefore, genetic analysis in tetraploid potato requires a genotyping system that can distinguish among alleles and quantify the allele copy number. In tetraploid species, the possible allele copy number (zygosity) categories include nulliplex (0), simplex (1), duplex (2), triplex (3), and quadruplex (4).

Several studies have shown that direct resequencing of amplicons by Sanger sequencing is sufficiently quantitative to allow the simultaneous discovery and genotyping of sequence variants in polyploids [8], [9]. Amplicon sequencing is a reasonable method to analyse a single or small number of target gene(s), but beyond that scale, more effort is required to design unique primers and optimize PCR parameters to ensure equal amplification of all alleles. Furthermore, in species like potato that exhibit high nucleotide diversity, the many indels usually create uninterpretable sequence reads that further reduces the throughput of Sanger-based amplicon resequencing. With the introduction of the next-generation massively-parallel sequencing (MPS) technologies, several novel approaches have been developed to discover, sequence and genotype not tens, but hundreds to thousands of genes simultaneously [2], [10]–[14]. Similar to Sanger sequencing, MPS can be used directly to genotype sequence variants. Using the former technology, genotype data are retrieved from chromatogram peak intensities at variant positions. For MPS, accumulation of sequence reads provides allele copy number estimates for each variant. Accuracy of this allele copy number estimate is dependent on sequencing depth. Genotyping-by-sequencing (GBS) of tetraploids will require higher read depth compared to diploids. In humans, for example, a sequence read depth of 30–35× is considered appropriate for accurate genotyping [15]. In order to identify each allele in tetraploids a simulation model suggested a read depth of at least 15× [13]. However, accurate genotyping (*p* = 0.95) of an autotetraploid organism implies also to discriminate digenic-simplex (*aaab*) as opposed to digenic-duplex (*aabb*) zygosity. Based on a binominal distribution this will require a sequence depth of at least 48×.

To achieve an increase in read depth, the portion of the genome that is sequenced can be reduced by applying, for example, RNAseq [2], [16], restriction enzyme based complexity reduction [17], [18], or sequence capture methods such as SureSelect, Nimblegen, and Raindance [19], [20], [21]. RNAseq is less suitable for GBS, since alleles may vary in transcription level across genes, tissues and stages, and thus may generate inaccurate genotyping data. Methods based on restriction enzyme treatments, on the other hand, are more likely to target non-coding parts of the genome, producing less useful data for functional gene analysis. Furthermore, restriction-based methods cannot target specific regions of interest, and nucleotide variants in the restriction site may interfere with digestion and cause null alleles. Sequence capture methods like SureSelect use oligonucleotide baits designed to bind to regions of interest, which can be specifically selected and enriched before sequencing [22]. For example, whole exomes [23] or regions associated with particular traits [19], [24] can be targeted. Sequence capture approaches require *a priori* availability of sequence data to design DNA capture probes. The recently sequenced genome of *Solanum tuberosum* group Phureja is appropriate for this purpose in potato [25].

Here we describe DNA resequencing results obtained from dozens of autotetraploid potato cultivars and one monoploid accession after genome complexity reduction using hybridisation-based in-solution enrichment. We subsequently used this data to identify sequence variants within and across cultivars and to call the genotypes of resequenced individuals. The accuracy of genotyping-by-sequencing was validated using a SNP genotyping assay. The resulting marker dataset is useful for describing allele frequencies, nucleotide diversity, and population structure in potato, and for validating QTLs via association analysis. Our approach is an efficient means of producing data for the design of both high and low-density SNP genotyping assays applicable to a wide range of potato cultivars, and the resulting tools can be used to address questions in population genetics and marker-trait association research.

## Materials and Methods

### Design of the Capture Library

A custom-designed SureSelect capture library containing 57,054 RNA oligonucleotide baits of 120-bp length each was developed. Genes targeted for enrichment were selected from the PotCyc metabolic pathway database [26], the Potato Maps and More database (PoMaMo) [27], an in-house QualitySNP marker database [28], [29], and a subset of single-copy genes homologous to the Conserved Ortholog Set II (COSII) [30]. Functional genes widely used as genetic markers for carbohydrate metabolism [31] and secondary metabolism [32]–[34], and a number of additional putative candidate genes for potato quality traits, were also included as targets. In addition to these functional genes, a number of intergenic sequences corresponding to AFLP markers, and a number of chloroplast and mitochondrial genes were included (Table 1). To avoid intron spanning baits initial cDNA targets were aligned to the *S. tuberosum* Group Phureja DM whole genome assembly v.1 and baits were designed based on the genomic reference sequence with highest homology. Genome annotation for the DM sequence was not yet available at the time the baits were designed, so genomic coding regions and intron/exon boundaries were estimated using GeneSeqer [35]. Other reference sequences used for bait design included the chloroplast genome of *Solanum tuberosum* cv. Desiree (NC008096), *Solanum tuberosum* mitochondrial sequences (S66866, X74826, X80386, X83206, X93576) and sequenced BAC clones of genotype RH89-039-16 [25].

**Table 1:** Targets for SureSelect bait library development.

	*Source*	*Genes*	*Target Contigs*	*Target Sequences*	*# of Baits (1 bait/20 bp)*	Proportion of baits in library
Nuclear genome	COSII database	248	853	439.6 kb	17,618	30.9%
	PotCyc database	149	467	142.5 kb	4,940	8.7%
	PoMaMo & Candidategenes	116	523	283.1 kb	11,481	20.1%
	In-house database	249	789	424.7 kb	17,174	30.1%
	AFLP sequences	45	202	87.8 kb	3,400	6.0%
Sub-total		807	2,834	1377.7 kb	54,613	95.8%
Organelle genome	Chloroplast	64	99	55.9 kb	2,272	4.0%
	Mitochondrial	4	12	4.7 kb	169	0.3%
	**Total**	**875**	**2,945**	**1,438.3 kb**	**57,054**	**100%**

For most targets, (cDNA) sequences were aligned to the potato reference genome, and the genomic sequence of each locus was used to design the baits.

During bait design, we aimed to optimize the in-solution hybridisation enrichment by avoiding targets exhibiting repeat elements and paralogous sequences in the reference genome, which can affect target-bait hybridization during enrichment and add difficulty to read mapping after resequencing. Stretches of repetitive sequences within the target regions were excluded from bait design using RepeatMasker (http://www.repeatmasker.org). BLAST homology search against the *S. tuberosum* Group Phureja DM whole genome assembly v.1 [25] was conducted to avoid the use of targets with paralogous and/or duplicated sequences. Except for a small number of target gene families of specific interest (e.g. polyphenol oxidases), target sequences having a secondary hit with E-value <10^−10^ were excluded from bait design. Regions consisting mainly of introns (>200–1000 bp) were avoided as targets. For each gene target sequence, we used an average of 3–4 continuous regions (contigs) for bait design, each region having an average length of 475 bp and, where applicable, including both exons and introns. OligoTiler [36] was used to tile the reference strand of each target region, with baits (of 120 bp in length) starting approximately every 20 bp. This produced a 6× bait tiling coverage and resulted in 57,054 unique baits for the SureSelect capture library (ELID 0274451). In total, the library targets 2,945 contigs (1.44 Mb, GC-content 39%; Table 1). Complete lists of sequencing targets and oligonucleotide bait sequences are available in XLS-file S1 and FASTA-files S1&S2.

### Plant Collection

A subset of 83 tetraploid potato cultivars (Table A in Supporting Information S1) was selected from a larger collection [37] using marker-based genetic distance estimates. The panel represents the global gene pool of commercial potato, both heirloom and contemporary, with emphasis on cultivars with high value in breeding and/or use. We also included a monoploid potato clone DM 1–3 511, derived by anther culture of the heterozygous diploid *S. tuberosum* group Phureja, clone BARD 1–3 of accession PI225669 [38]. The DM 1–3 511 clone is highly related to the recently sequenced clone DM 1–3 516R44 (CIP801092) [25], a doubled monoploid derived from the same BARD 1–3 clone [38].

### Extraction and Fragmentation of DNA

DNA was extracted from leaves ground in liquid nitrogen using KingFisher Genomic DNA Purification Kit (Thermo Scientific) and the KingFisher Ml magnetic nucleic acid extraction system (Thermo Scientific) according to the manufacturer’s procedures. DNA concentrations were quantified with a NanoDrop ND-1000 spectrophotometer (Thermo Scientific) and diluted to 35 ng/µl. Of each DNA sample, 3.5 µg was fragmented by Adaptive Focused Acoustics on a Covaris S2 instrument (Covaris, Inc.), using a 10% duty cycle at intensity 4 for 120 seconds with 200 cycles per burst. Fragmentation of DNA to an average size of about 300 bp was verified using Bioanalyzer High Sensitivity DNA chips (Agilent).

### Indexed Sequence Library Preparation

Custom-ordered, HPLC-purified, indexed adapters consisted of complementary Illumina adapters PE1 and PE2 [39], with PE1 extended by a 4-bp index sequence and an extra terminal T to facilitate sticky-end ligation. The reverse (PE2) adapter was extended by the reverse complement of the PE1 index (Table B in Supporting Information S1). The twelve indices had a balanced base composition and a minimal edit distance (i.e. the number of mutations required to change one index to another) of 2 bp to detect sequencing errors in the index region. To pair the adapter strands, mixtures of the forward and reverse strands (each 50 µM in TE) were incubated at 95°C for 2.5 minutes, followed by a cool down (−1°C/30 sec.) to 25°C and subsequently diluted to a working concentration of 12.5 µM. DNA sequencing libraries were prepared using the NEBNext DNA Sample Master Mix Set 1 (New England Biolabs). Purifications were carried out between end-repair, dA-tailing, and adapter ligation steps using AMPure XP beads (Agencourt Bioscience). To index the 84 potato DNA extracts, each of the twelve unique adapters was ligated to seven different DNA samples by mixing adapter and DNA in a molar ratio of approximately 20∶1. After the initial ligation of 15 minutes at 20°C, samples were held at 4°C overnight and purified using AMPure XP beads. Adapter ligation was verified by fragment size analysis using Bioanalyzer High Sensitivity DNA chips.

### In-solution Hybridization and Target Enrichment

The 84 indexed paired-end sequencing libraries were hybridized to the SureSelect capture library according to the manufacturer’s instructions (Agilent SureSelect Target Enrichment System for Illumina Paired-End Sequencing Library Protocol, Version 1.0 May 2010), with minor modifications. Size selection on gel was omitted, allowing us to skip the standard pre-amplification step prior to in-solution enrichment and reduce the number of clonal reads in the later generated sequences. The use of index-specific blocking oligos to reduce non-specific pull-down due to adapter-adapter hybridisation can also be avoided when pre-amplification is excluded, as non-amplified Y-adapters do not concatenate. We therefor excluded from the hybridization mix Block #1 (Human C0t-1 fraction) [40], which is irrelevant for plants, and excluded Block #3 (PE adapter block) [40]. We used half of the specified hybridization volumes. Of each indexed DNA sample, 50–400 ng was mixed with 1.25 µg salmon sperm DNA (Block #2), denatured, and hybridized to 100 ng SureSelect biotinylated RNA baits developed from the capture library. The hybridization mix was held at 65°C for 24 h for hybridization, then added to 250 ng (25 µl) T1 streptavidin Dynabeads (Invitrogen), and pulled down. Bait-selected DNA was purified using AMPure beads, and amplified for 14 cycles (Tm 60°C) using 1 U Herculase II Fusion proofreading DNA polymerase (Agilent) and 25 µM each of custom primers PE1.0 and PE2.0 (Table C in Supporting Information S1). The alternative DNA polymerase was chosen after the polymerase included in the NEBNext DNA Sample Master Mix Set 1 was seen to cause a negative shift in average fragment size.

Amplified, enriched DNA libraries were purified using AMPure XP beads and evaluated by agarose gel electrophoresis, NanoDrop, and Bioanalyzer High Sensitivity DNA chips. Library subsamples were also quantified by qPCR using a two-step amplification protocol [95°C activation for 2 min, 40× (95°C 10 sec., 60°C 30 sec.)] and primers qPCR_1.1 and qPCR_2.1 (Table D in Supporting Information S1).

### Hi-Seq2000 Sequencing and Data Preprocessing

Seven distinct resequencing pools were made by combining equimolar amounts of twelve unique, differently-indexed, enriched DNA library samples (Table A in Supporting Information S1). Pooled libraries were sequenced on a Hi-Seq2000 (Illumina) lane using 100-base paired-end sequencing at the Genome Analysis Facility of the University Medical Center Groningen (UMCG). Four sequenced pools that generated low cluster numbers and/or partly failed reverse read sequences were repeated. Initial quality checks (average read quality per cycle, average read quality, base call % per position) were performed using FastQC (http://www.bioinformatics.babraham.ac.uk/projects/fastqc/). Sequences in each pool were categorized to their biological sources according to the 4-bp index using NovoBarCode (Novocraft) with the average edit distance set to 3. The first 5 bp of each de-multiplexed sequence read (i.e., the 4-bp index plus an extra T used for ligation) were removed.

Sequence read-pairs containing ≥63 nucleotide sequences with 100% match to chloroplast genome sequences of *Solanum tuberosum* cv. Desiree (NC008096) were extracted from the de-multiplexed sequences and kept for separate analysis. Remaining sequence reads were aligned using the Burrows-Wheeler Alignment tool (BWA) [41] with default alignment parameters, except for the maximum edit distance, which was relaxed to seven due to the expected high sequence divergence between potato alleles. Sequences were aligned to the annotated superscaffolds (DMGv.3.4, comprising 705.5 Mb of sequence in 1,419 superscaffolds and 43 Mb of non-ACGT bases) of the *S. tuberosum* Group Phureja DM whole genome assembly v.3 reference genome [25]. The 120-bp SureSelect bait sequences of genomic origin were correspondingly mapped to the annotated superscaffolds, and baits with a mapping quality (MQ) ≥37 were used to define the genomic target regions.

Alignment data was processed with SAMtools and Picard [42] to mark duplicate reads and estimate the average insert size of the paired-end reads. The Genome Analysis Toolkit (GATK) [43] was used for indel realignment, base-score recalibration, and extraction of read depth information. Read depth and coverage data were processed with custom Perl scripts and BEDTools [44]. The raw sequencing reads and mapping assembly are available on our server (http://datarelease.plantbreeding.nl/).

### Sequence Variant Detection and Genotype Calling

For covered regions (see Results for definition), sequence variants were identified simultaneously among the aligned reads from all 83 tetraploids and the single monoploid using the FreeBayes polymorphism discovery algorithm [45]. Sequence variants included binary SNPs, MNPs, and small indels, as well as allelic series of tri-SNPs and tetra-SNPs, multi-allelic MNPs, and indels with a variable number of (repetitive) nucleotides. Reads marked as duplicate, with more than seven base mismatches, more than three separate gaps, or with MQ<30 were excluded for variant calling. The expected mutation rate or pairwise nucleotide diversity was set to 0.01. In order to include an alternate allele as a variant, supporting bases required a minimum base quality (BQ) of at least 13, and at least one supporting alignment was required to have BQ≥20. Furthermore, the alternate allele had to be observed in at least 5 reads and represent at least 12.5% of the observations of reads at that locus within a single potato sample. Mapping quality of alleles was included when calculating posterior probabilities, and variants were only called for sites that had a probability of polymorphism greater than 0.95. Sequence variants adjacent to indels, which were likely due to local misalignment, were filtered using GATK [43].

For genotype calling, zygosities of all sequence variants were resolved by allele-specific read depths for all non-duplicated reads with MQ≥13 using FreeBayes. This resulted in nulliplex (*aaaa*), simplex (*aaab*), duplex (*aabb*), triplex (*abbb*) and quadruplex (*bbbb*) genotype calls relative to the reference sequence for the tetraploid potato samples, while variants in the monoploid sample were genotyped as either absent or present. Hence a variant is called not only if a genotype differs from the reference, but also if a genotype differs from any other genotype. Only variants previously identified by variant calling were used for genotyping, and samples required a minimum read depth of 15× at the variant position to yield a genotype call. In addition to this 15x coverage threshold we applied a threshold GQ≥26. Genotyping Quality (GQ) was calculated by a likelihood estimate to determine the probability that a genotype call was different from the true genotype using FreeBayes. It was encoded as a phred quality score (−10×log_10_(*p*)) and included read depth at a variant position as a parameter.

### Diversity Analysis and Prediction of Functional Consequences of Allelic Variants

To calculate the nucleotide diversity, we first calculated the gene diversity (heterozygosity) of each binary variant; GD = 1−ΣP_i_^2^, where P_i_ is the frequency of the *i*th allele and GD is summed across *n* alleles. Nucleotide diversity (π) was calculated by averaging GD over all nucleotide sites – or all coding and/or non-coding nucleotides sites – on a contig or gene.

The functional effect (codon mutations, splice site mutations, frame shift mutations) of sequence variants was predicted using snpEff [46] and gene annotation DMGv3.4 of the DM genome.

In order to identify population structure, principal components analysis (PCA) was performed on genotype scores using the FactoMineR library of R [47]. Only genotype scores of variants called in all 84 cultivars were included. Since all genotype scores were measured in units of allele copy number, the data were not scaled. K-mean clustering of the first three principal components was used to identify genotype clusters.

### Association Analysis

Adjusted phenotype means for plant maturity and tuber flesh colour in each of the 83 tetraploid cultivars, measured previously over a period of five years [37], [48], were used as trait values for conducting association analysis. Additive and dominant genotype models were each tested both with and without correction for population structure. The genotype clusters identified by PCA analysis were used as the adjustment factors for population structure. For dominant association models, linear regression models were used as implemented in PLINK [49], with tetraploid data recoded into diploid homozygous/heterozygous scores, using binary allelic variants only. Adjustment of p-values to correct for multiple testing effects was carried out using step-up FDR control as implemented in PLINK. For the additive tetraploid genotype models, we applied linear regression models implemented in Genstat. For each trait and each marker the model fitted was: response = allele copy number (+ structure)+error.

### Validation of Genotype Calling

The accuracy of GBS genotype calls was validated using the Kbioscience Allele-specific Polymorphism Assay (KASP) SNP genotyping platform. Binary SNPs identified in the sequence data that exhibited a minor allele frequency between 0.15 and 0.35 were selected as candidate for assay design. To assure independence among all SNPs in the KASP assay (i.e., use of a unique SNP from each haplotype block), SNP data from GBS were clustered using hierarchical cluster-analysis, and a single SNP from each cluster having a correlation coefficient of *r* ^2^≥0.16 was used for the KASP assay, yielding 768 SNPs in the final design. We KASP-assayed DNA from 65 potato cultivars included in GBS, with two of these cultivars measured in duplicate to assess KASP genotyping consistency. A number of additional diploid potato clones were assayed (∼96) and used to examine the signal ratio of the nulliplex, duplex and quadruplex genotype signals. The software package fitTETRA [50] was used for full tetraploid zygosity genotype calling. In total, 270 of the 768 KASP assayed SNPs were selected for validation of the GBS calls. This selection was based on (1) sharp clustering of the signal ratios of discrete genotype classes, (2) clustering of signal ratios of heterozygous diploids with duplex tetraploids, and (3) clustering of signal ratios of homozygous diploids with nulliplex or quadruplex tetraploids.

## Results

### Sequencing and Mapping of Enriched Libraries of 84 Potato Genomes

**Figure 1:**
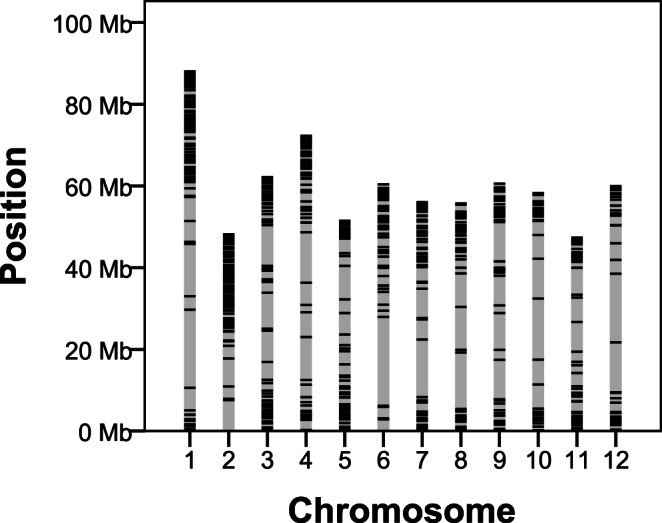
Mapping of resequencing targets onto the potato genome. Physical position of the approximately 800 target genes scattered across the 12 potato chromosomes. Physical positions are ordered according to the latest pseudomolecule order (v.3 beta). The distribution of the ∼800 genes reflect the physical proportions of gene rich chromosome arms and gene poor heterochromatic regions.

We designed an in-solution hybridisation capture library targeting primarily introns and exons of nuclear coding genes, but also including intergenic, chloroplast, and mitochondrial sequences. Baits targeting nuclear genome sequences in the enrichment library covered approximately 1.3 Mb of the potato DM reference genome sequence, scattered across all 12 chromosomes (Figure 1 & BED-file S1).

Genomic DNA sequencing libraries from 83 diverse tetraploid potato cultivars and progenitor lines, and a single monoploid potato clone, were indexed by cultivar with 12 distinct 4-bp index sequences and individually enriched using the capture library. Indexed and enriched samples were multiplexed in pools of 12 unique indices and paired-end sequenced. In total, 592,100,112 read-pairs were obtained, representing approximately 100 Gigabases of sequencing data. The cultivar-specific sequence index could be identified in 96% of the read-pairs (Figure A in Supporting Information S1). For 19% of the read-pairs, cultivar identification was based on the index from only one read of the pair. In most cases, this resulted from technical failure of the reverse-read sequencing.

Within each multiplexed pool, the cultivar-specific read counts rarely reached a twofold difference between cultivars, indicating approximately equivalent amounts of DNA were added to a pool from each cultivar present. Initial inspection of the cultivar-tagged sequences showed that chloroplast-derived sequences were highly abundant (60% of the read-pairs). These sequences were filtered using broad homology criteria and saved for a later analysis of its own. The remaining 227,263,706 read-pairs were aligned to the DM reference genome, with 80% mapping. This resulted in 23.9 Gigabases of high quality (MQ≥13) genome-aligned sequence data. Among mapped reads, 8% were marked as duplicate reads aligning with identical start and end positions on the reference genome.

### Genome and Target Coverage

As a consequence of the enrichment method sequences aligned not only to target regions but also to flanking and off-target regions. To define the “accessible” part of the genome, i.e., the part with adequate coverage to allow identification of sequence variants, we used the following criteria: (1) at least five cultivars had to be sequenced at the base position, with a threshold criterion of at least 20 component reads with MQ>30 per cultivar; (2) in view of the fragment sizes of mapped reads (261±94 bp, mean±SD), regions defined as covered were at least 261 bp long; (3) regions were discarded when more than 5% of reads within the region aligned to multiple locations with equal probability (MQ = 0); and (4) finally, regions with >80% homology to chloroplast and mitochondrial sequences were removed. This resulted in 2,445 sequenced contigs with a total length of 2.1 Mb (XLS-file S2 and BED-file S2). A total of 12.5 Gigabases of high quality sequence data aligned to these contigs, providing an average read depth of 5,871× when all cultivars were considered together. Median depth per cultivar within this accessible genome was 63×, ranging from 15× to 177× (Table 2 and Table A in Supporting Information S1).

**Table 2:** Summary of target enrichment sequence coverage for 84 potato cultivars.

*“Accessible” genome parameter*	*Value*
Number of covered regions (contigs)	2,445
Sequence length	2,136,143 bp
Coding sequence (DMGv.3.4)	655,930 bp
Sequencing depth per cultivar at contigsb,c	70±36× (Median 63×)
Sequencing depth per cultivar at targetsequenceb,c	95±48× (Median 88×)
Genes covered	977
Target genes covered	793 out of 807
Target sequence covered	1,294,097 bp (97%)^a^
Target+directly flanking sequences	1,848,192 bp
Off-target sequence	287,951 bp (13%)

a Percentage of target bait sequence mapped to the DM genome.

b Only sequenced nucleotides that aligned with high quality (MQ≥13).

c Mean and standard deviation.

Almost all genomic regions and genes targeted by the enrichment library fell within the accessible regions, with 97% of target sequences and 793 of 807 nucleartarget genes covered. In total, 10.7 Gigabases of high quality sequence data aligned to target regions with a median depth per cultivar of 88×, ranging from 20× to 240×. Accessible flanking regions had an average length of 150 bp and comprised 554 kb of additional sequence. Regions flanking target sequence but interrupted by poor coverage for a small number of nucleotides, and more remote off-target regions, accounted for 288 kb (13.5%) of the accessible genome.

### DNA Sequence Variants

A total of 129,156 putative sequence variants (SNPs, MNPs and indels) were identified in the accessible genome (Table 3, CSV-file S1, and VCF-file S1). The density of substitution variants (SNPs and MNPs) was 1.6 times higher in non-coding regions than in coding regions, and the indel density was 12 times higher in non-coding regions. The transition/transversion ratio (T_s_/T_v_), calculated only for biallelic SNPs, was 1.55, and the ratio of non-synonymous to synonymous SNPs (pN/pS) was 0.64.

**Table 3:** Overview of DNA variants observed across 84 cultivars in the accessible potato genome.

	*Number of sequence variants called*		
*Variant type*	*Accessible genome (2136 kb)*	*Non-coding (1480 kb)*	*Coding (656 kb)*
Di-nucleotide SNPs	105,812	84,454	25,358
Tri-SNPs	5,304	4,097	1,207
Tetra-SNPs	96	66	30
Indels	13,094	12,641	453
MNPs	4,850	4,084	766
***Total***	***129,156***	***101,342***	***27,814***
*Average variant density*	*1/16.5 bp*	*1/14.6 bp*	*1/23.6 bp*

Across all cultivars, an average variant density of 1/24 bp in coding regions and 1/15 bp in non-coding regions was observed. Within a single tetraploid cultivar, on average 52,233 sequence variants (1/42.5 bp) were observed. On average each cultivar had 116 cultivar-unique variants, ranging from 0 to 2,688. Cultivars like Vitelotte Noir, the only cultivar with purple flesh colour in our samples, and those with wild species introgression segments contained a relatively high number of cultivar-unique variants (e.g., up to 2.0% of all variants within cv. Vitelotte Noir). Cultivars without unique variants either had ancestors that were widely used in breeding of novel potato cultivars or had themselves been used for this purpose (e.g., cv. Agria and cv. Katahdin respectively).

**Figure 2:**
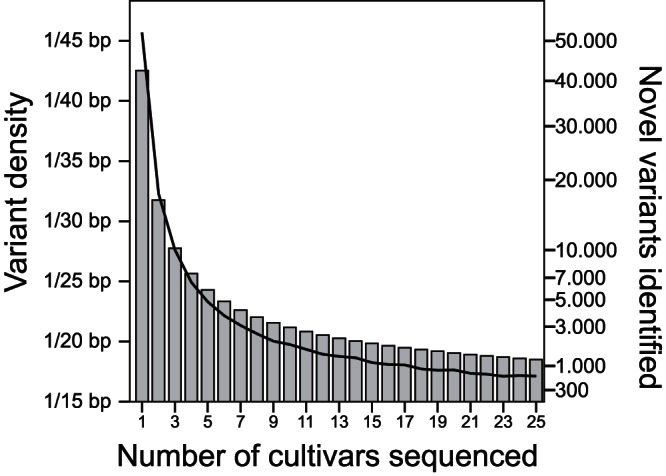
Sequence variant density as the number of randomly-added cultivars increases. The bars show variant density (primary Y-axis), and the black line shows the number of newly-identified variants (secondary Y-axis) as a function of the number of sequenced cultivars. Data is not shown after the 25^th^ cultivar, but continues to drop to a variant density of 1/16.4 bp and an average of 116 novel variants at the 84^th^ cultivar.

To evaluate the increase in variant density per additional sequenced cultivar, we permutated the order of the cultivars a thousand times and calculated the variant frequency at each incremental step (Figure 2). More than half of all variants were detected by selecting three random cultivars. When 16 random cultivars were selected, 84% of all variants were detected, and the number of novel variants that could be identified by sequencing an additional cultivar dropped below 1% of the variants already discovered. To detect 95% of all the identified variants, 46 random cultivars were required.

### Genotype Calling and Allele Frequencies

**Figure 3:**
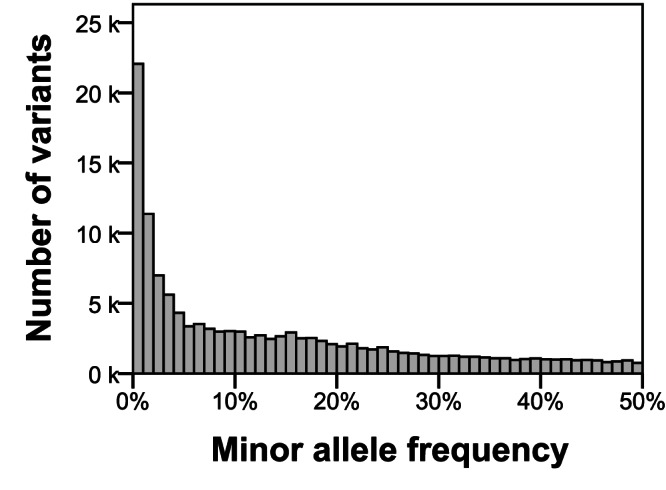
Distribution of minor allele frequencies (MAF) of all 129,156 genotyped sequence variants.

To be assigned a valid genotype call for a specific variant position, a cultivar required a minimal read depth of 15× at that position. Using this criterion, 86.6% of all possible genotype calls were valid (i.e., of the matrix of 84 cultivars by 129,156 sequence variants, 13.4% had insufficient read depth to make a call), equivalent to a per-locus average of 73 out of 84 cultivars receiving a genotype call. For 42,625 sequence variants (33% of all identified sequence variants), all 84 cultivars were genotyped, and more than 90% of all sequence variants were genotyped in at least half of the cultivars. Population-level allele frequencies were calculated using all valid (≥15×) calls. The distribution of minor allele frequencies (MAF) is shown in Figure 3. The average MAF was 0.14; 17.4% of all sequence variants had MAF<0.01, and 60.9% had MAF>0.05. For 13,458 sequence variants, 10.4% of all identified, the allele in the DM reference genome differed from the major allele in the population.

### Nucleotide Diversity

**Figure 4:**
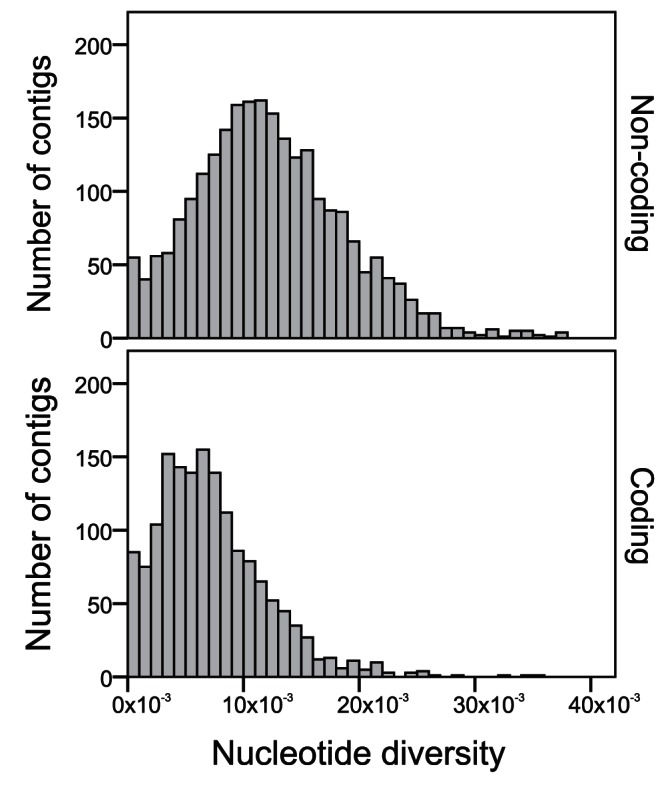
Distribution of nucleotide diversity across sequenced contigs for coding and non-coding regions.

**Figure 5:**
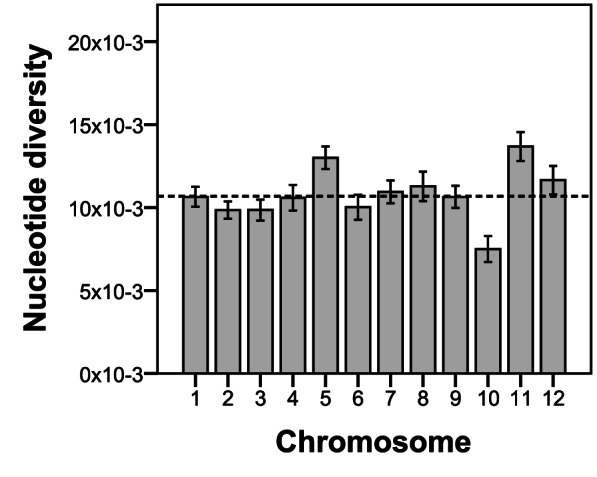
Mean nucleotide diversity across sequenced contigs per chromosome. Dashed line represents genome wide nucleotide diversity (both coding and noncoding sequences), and error bars represent 95% confidence intervals.

We next calculated the nucleotide diversity (π) for each of the 2,445 sequenced contigs, for each gene, and for each chromosome. Mean π of the covered genome was 1.07×10^−4^ (Table 4). As expected due to functional constraints on evolutionarily tolerable mutations, π was lower for coding regions than for non-coding regions (Table 4 and Figure 4). The physical position of contigs on the potato pseudomolecules was used to plot π over the twelve potato chromosomes (Figure B in Supporting Information S1). Mean nucleotide diversity for chromosomes 5 and 11 was significantly higher relative to other chromosomes (Figure 5). In contrast, the mean π for chromosome 10 was significantly lower than for other chromosomes. Individual genes with low nucleotide diversity were observed on all chromosomes (XLS-file S3).

**Table 4:** Nucleotide diversity in potato (mean value and standard deviation across contigs).

Nucleotide diversity (×10-3)	10.7±5.4
Nucleotide diversity (×10-3), coding sequence	7.3±4.8
Nucleotide diversity (×10-3), non-coding sequence	12.4±6.7

### Population Structure

**Figure 6:**
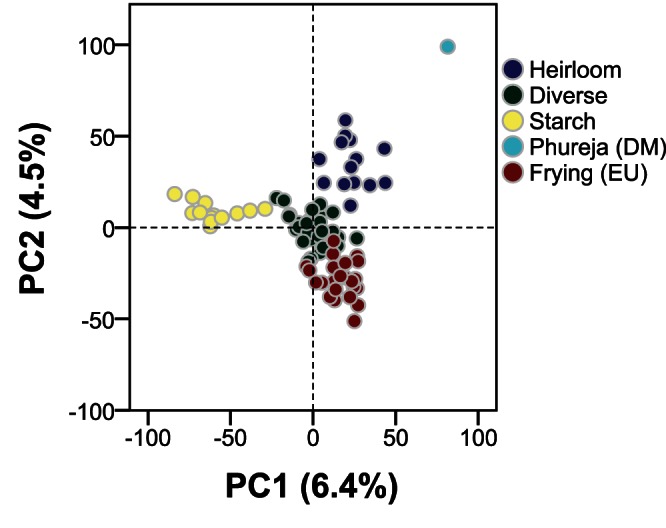
First and second components from principal component analysis of potato sequence variant genotypes. Population structure was analysed using ∼43 K sequence variants genotyped in all 84 cultivars. The first three components describe 14.7% of the variance. Based on these three components, the cultivars were clustered into five groups. The most distant cultivar is the monoploid *S. tuberosum* Group Phureja clone**.** In the centre of the PCA plot cultivars of diverse, world-wide origins are observed. Three additional divergent groups can be observed, consisting of heirloom cultivars, frying cultivars from continental Europe and cultivars and germplasm used in starch industry.

Population structure was analysed using the sequence variants that were genotyped in all 84 cultivars. The first three components of a principal component analysis described respectively 6.4%, 4.5% and 3.8% of the variance. In the centre of the PCA plot, a cluster of cultivars of diverse origin was located (Figure 6). Three groups diverging from this set of cultivars were noted, which consist of (a) heirloom British cultivars, (b) a number of typical frying cultivars from continental Europe, and (c) progenitors of potato cyst nematode (PCN) resistance and cultivars bred for the starch industry with resistance to PCN. The heirloom cultivar group is closest to the more distant monoploid *S. tuberosum* Group Phureja clone, which fell into a cluster of its own.

### Association Analysis

**Figure 7:**
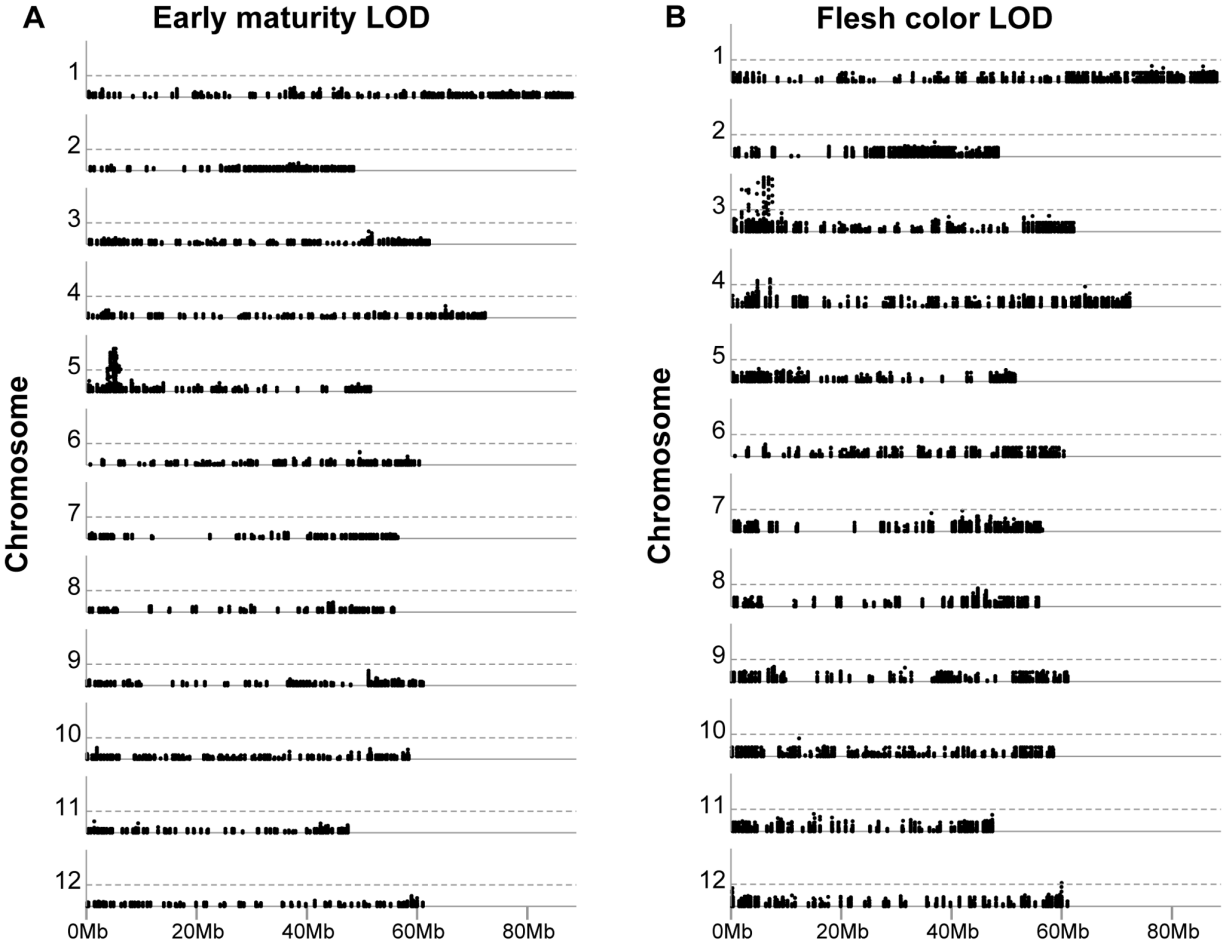
Manhattan plots of *p* values for associations between DNA sequence variants and two phenotypic traits in potato: (A) plant maturity and (B) tuber flesh colour. The FDR corrected −log_10_(*p*) values from GWAS analysis are plotted relative to the physical position on each of 12 potato chromosomes. The horizontal dashed line is plotted at the FDR-corrected significance threshold of α = 0.001 (−log_10_(*p*) = 3).

The genotype dataset was used for a genome wide association study (GWAS) to validate the sufficiency of this data for identifying known QTLs for plant maturity and tuber flesh colour. Association analysis was performed using both additive and dominant genotype models with separate tests with and without correction for population structure. To a large extend, the results of the dominant and additive models overlapped. The results of the dominant allele models are shown in Figure 7. Even with the small population size of 83 phenotyped cultivars, the well-known QTL for early plant maturity on potato chromosome 5 was clearly detected (−log_10_(*p*) = 6.0) and explained 44% of the observed phenotypic variance. Using the current data, this QTL mapped to a region of approximately 371 kb within superscaffold PGSC0003DMB000000192 containing 28 strongly associated variants (−log_10_(*p*)≥5). For tuber flesh colour, a major QTL was observed on chromosome 3, mapping to a region of approximately 683 kb containing 27 strongly associated variants (−log_10_(*p*) ≥7). These sequence variants were located within and near *CHY2* (*β-carotene hydroxylase*), a well-known gene influencing flesh colour via carotenoid synthesis, and explained 61% of the phenotypic variance. Two additional minor-effect QTLs for flesh colour were found on chromosomes 4 and 12. When corrected for population structure, the QTL on chromosome 12 was not significant. The flesh colour QTL on chromosome 4 (−log_10_(*p*) = 3.7) explained 9% of additional phenotypic variance beyond that explained by the major QTL.

### Validation of Genotyping-by-sequencing Data

A subset of 270 binary SNPs was selected to validate genotype calls made by GBS using KASP genotyping assays. For one of the two cultivars duplicated in the KASP assay, all genotype calls were identical between replicates, and for the other cultivar, only two genotype calls varied between replicates. The expected KASP genotyping error rate of this subset of SNPs is thus very low (0.4%). For concordance analysis, expected genotype calls were obtained from these 270 KASP assay SNPs and observed genotype calls were obtained by the GBS results. Scored as either homozygous (i.e., nulliplex or quadruplex) or heterozygous (i.e., simplex, duplex, or triplex), 97.9% of the homozygous and 99.9% of the heterozygous GBS calls were concordant with the results of KASP genotyping. When heterozygous GBS calls were split into simplex, duplex and triplex categories, overall concordance with KASP genotyping dropped to 94.4%. For duplex calls, which were most difficult to classify, only 90.3% of the calls were concordant. We therefore applied a genotype quality (GQ) filter to validate GBS/KASP concordance in the five discrete zygosity classes. A threshold of GQ≥26 was required to reduce the number of discordant duplex calls below 5%. Applying the GQ26 filter to the complete set of 129,156 sequence variants yielded 74.8% of variants with an assigned genotype call, with an average of 63 out of 84 cultivars genotyped per variant position. This is equivalent to 25.2% of unassigned genotype calls, yielding approximately two-fold more unassigned genotype calls compared to the set subject only to the 15× read-depth threshold. Median read depth of all GBS genotype calls meeting the GQ26 threshold was 61×, and for duplex calls this was 81×. Overall concordance between the GQ26 filtered set and KASP genotyping was 98.4%, with 96.2% concordant duplex calls (Table 5).

**Table 5:** Concordance between genotyping-by-sequencing and KASP genotyping calls.

	*Expected genotype call (KASP)*					
*Genotyping-by-sequencing*	*Nulliplex*	*Simplex*	*Duplex*	*Triplex*	*Quadruplex*	*Total*
Concordant calls	4,975	4,861	2,204	1,373	1,272	14,685
Discordant calls	67	25	86	34	25	237
Percentage of concordant calls	98.7%	99.5%	96.2%	97.6%	98.1%	98.4%
Median read-depth of concordant calls	85×	91×	101×	92×	75×	89×
Median read-depth of discordant calls	52×	56×	73×	83×	47×	61×
Percentage of alternative reads for concordant calls	0%	23%	49%	76%	100%	–
Percentage of alternative reads for discordant calls	4%	36%	34%	56%	97%	–

The genotype calls derived from each method for 270 binary SNPs were compared. Sequencing calls were filtered by a minimum read depth of 15× and a minimum genotype quality score of GQ26.

### Analysis of Chloroplast Reads

**Figure 8:**
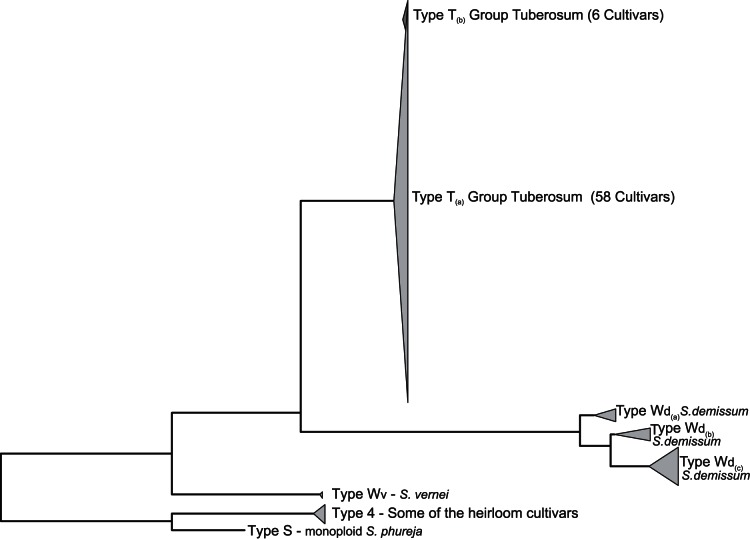
Neighbour-Joining tree of chloroplast haplotypes. The distances of 241 sequence variants for the 84 cultivars were computed using the Jukes-Cantor method and the tree inferred using the Neighbor-joining method.

As an initial analysis of chloroplast reads, 100,000 paired-end reads per cultivar, with index sequences on both sides, were mapped to the chloroplast reference genome. A total of 241 sequence variants, covering the chloroplast genome, were identified (VCF-file S2). Since chloroplast sequences are monomorphic, the chloroplast haplotypes could be directly inferred. Four main chloroplast types, with a number of sub-types, were found using a phylogenetic approach (Figure 8 and Table A in Supporting Information S1). A number of cultivars contained distinct chloroplast genomes resembling those of *S. demissum*, *S. vernei* and *S. tuberosum* Group Phureja, but most resembled those commonly found in *S. tuberosum* Group Tuberosum cultivars.

## Discussion

### Target-enriched Genome Sequencing

This paper presents evidence that GBS in complex polyploid genomes can be performed accurately, especially with regard to the specific requirement of accurate allele dosage detection. Below, we will argue that our method of target enrichment was of great importance for genotyping accuracy. Target enrichment allowed the achievement of sufficient read depth. Furthermore, bait-hybridisation enrichment allowed exclusion of complex multi-copy or repetitive regions of the genome, and avoidance of potential difficulties in distinguishing allelic variants from paralogous sequences [51]. We focussed on a single copy genes, e.g. from the set of conserved orthologous sequence genes (COSII) [30] and used uniquely mapped genes to define the genomic target regions. It is not excluded that individual potato genotypes may be polymorphic for specific gene duplications (Copy Number Variants). Indeed, for some genes read depths were observed twice or more than average in specific cultivars, suggesting gene duplications in these cultivars (data not shown). In these rare cases the duplication maps to the same location on the reference genome and could affect genotype calling. However, we feel confident that this is a relatively rare phenomenon, because the validation of genotype calling with KASP assays showed high concordance.

The SureSelect enrichment method yielded sequences of which 45% aligned to the target sequences. Other in-solution DNA enrichment studies in plant species have not yet been published, but this on target percentage is consistent with the 40–50% reached in human and animal studies that have used the SureSelect system for target enrichment [19], [40].

To reduce sequencing costs we multiplexed twelve cultivars in a sequencing pool. The custom index adapters have proven valuable as multiplex adapters; no index-specific bias in read counts was observed and over 96% of the generated reads were assigned to cultivars. The inclusion of these indices permits tracking of the alleles’ cultivar source so that zygosity can be determined in individual genotypes, allowing GBS of many individuals. Larger numbers of indices can be easily created [17] and allow multiplexing of tens to hundreds of individuals. We found consistent enrichment across all indexed samples, and virtually all target sequences were covered covered at a sufficient depth, with a median average read depth of 88× per cultivar for nuclear target sequences (63× for the extended “accessible” genome). The read depth of nuclear sequences was reduced by the large number of cpDNA reads (60% of total reads) generated in this study. This large share of cpDNA reads was unforeseen in view of the 4% share of cpDNA baits used for target enrichment. In retrospect, this large proportion is almost certainly due to the copy number variation between nuclear DNA and cpDNA. The latter is present in about 100 copies per plastid and about 100 plastids per leaf cell [52]. Hence, bait library design should avoid sequences from, or homologous to extra-nuclear DNA.

In the estimate of allele copy number we assume no ascertainment bias; i.e., the relative number of allele-specific sequencing reads is proportional to the allele copy number. However, in-solution hybridisation based target enrichment may bias the pool of captured DNA targets that are sequenced towards those variants which preferentially hybridize and have higher sequence similarity to the reference bait sequence [19]. Anticipating this bias, we used a high probe tiling redundancy (6×, one RNA bait every ∼20 bp) to reduce allele-specific bias during hybridization. Furthermore, we relaxed mapping quality settings for counting the reads mapped to the reference genome. In a preliminary genotype analysis performed after sequence variant calling, only reads with a very high mapping quality (≥MQ30) were used to determine the relative number of allele-specific sequencing reads. This caused a large ascertainment bias due to severe underrepresentation of non-reference alleles. Inclusion of reads with a lower mapping quality (MQ≥13) strongly reduced this ascertainment bias. When the GQ-filter was applied, 95% of the sequence variants were concordant, as shown by the KASP validation (Table 5). The average read ratio for these calls shows no bias. We conclude from this study that hybridisation based capture bias hardly affected genotype calling, even in potato germplasm where reference and non-reference reads often have only about 95% sequence homology. We have not examined the genotyping accuracy of MNPs or indels, but larger indels are likely to reduce capture and mapping efficiency.

We used a mapping approach to align the sequencing data to the potato genome sequence [25]. An advantage of alignment to an annotated reference genome is that it allows prediction of whether a sequence variant falls within or near a gene of interest, and whether it is expected to cause a functional change in the protein product (e.g. synonymous vs. non-synonymous) that might alter the enzyme activity of the protein. This can be very useful in determining whether a particular sequence variant is likely to be responsible for a phenotype of interest. We observed approximately twenty-five thousand non-synonymous variants, with 50 variants that cause premature stop codons in 43 out of 807 genes. Hence, natural diversity of potato offers a substantial resource for the identification of knockouts for gene-function studies.

A disadvantage of mapping sequence reads toward a reference sequence is that structural variation like chromosome rearrangements, inversions and large (transposon) insertions are likely to be missed. Structural variation between reads and reference genome complicates the mapping of reads. This disadvantage can be partly avoided by *de novo* assembly, but computational difficulties associated with the assembly of highly diverse polyploid species like potato makes mapping sequence reads to a reference sequence a more straightforward approach. In this study we did not systematically look for structural variants, nor did we consider gene copy number variants (CNVs). Methods for detecting both structural variants and CNVs using a mapping approach have however been developed [53], [54] and could be applied. Fortunately, structural variants can frequently be detected using sequence variants in linkage disequilibrium (LD). For example, orange potato flesh colour due to zeaxanthin accumulation, caused by a transposon insertion in the zeaxanthin epoxidase gene was initially detected using SNPs [34].

### Sequence Diversity Analysis

This study confirms the high sequence diversity of potato, albeit using a much higher number of potato cultivars and on a more genome-wide scale than previous studies [55], [56]. We found an overall frequency of one variant every 17 bp in the cultivar population, 1/15 bp in non-coding and 1/24 bp in coding regions. This is somewhat higher than the one variant per 21–23 bp reported in previous studies on potato [55], [56], reflecting the differences in the number of potato cultivars used. Compared to other crops where both coding and noncoding regions were analyzed for nucleotide diversity, total nucleotide diversity in potato cultivars (π = 10.7×10^−3^) is larger than that in sugar beet (7.6×10^−3^) [57], maize elite lines (6.3×10^−3^) [58], and soybean (1.25×10^−3^) [59]. Simko et al. (2006) presented a somewhat higher nucleotide diversity for potato of π = 14.6×10^−3^, but their sample included a number of distantly related wild potato species [56].

For discovery of common sequencing variants (i.e. variants with a high MAF), only a limited number of potato cultivars have to be sequenced, as more than half of all variants were already detected by sequencing three random cultivars. In a previous study in potato, SNPs have been identified in cDNA of a set of three to six – mainly North-American – potato cultivars, using Sanger EST-sequences available in GeneBank from three potato cultivars, and using high-throughput transcriptome sequencing on three additional cultivars [2]. The SNPs identified in this relatively small sample, are expected to mainly represent the common SNPs from all coding sequences, whereas this study included introns and sampled rare alleles. Based on exons sequenced in both studies an overlap of only 2,572 variants was expected between our 129,156 variants and the 69,586 mapped SNPs identified by Hamilton et al. (2011). We detected 2,362 (92%) of these SNPs. As only one cultivar was included in both studies (cv. Bintje), the 8% of variants undetected in our study might represent rare variants more specific to the North American cultivars predominantly sampled by Hamilon et al. (2011), or may be false positive/negatives in either study. As a result of the at least 14-fold larger sample size and larger geographic diversity targeted by our study, we find approximately 15,000 extra variants in the exon sequences covered by both studies. These include multi-allelic sequence variants at 6% of the base positions where SNPs were called by Hamilon et al. (2011). When these SNPs are assumed bi-allelic, they may complicate genotype calling in a SNP genotyping array.

An important feature affecting the application of sequence variants as molecular markers is their MAF, which influences the type of information provided by the marker in different populations. Moderate-frequency alleles are valuable for mapping studies, where it is desirable to maximize the number of polymorphic markers between two parental lines. Rare variants can, if not assayed, cause ascertainment bias in phylogenetic reconstructions, overestimations of mean diversity [60], and spurious correlations in association mapping [61]. This paper presents an L-shaped distribution of the MAF (Figure 5) which points to an abundance of rare alleles in the cultivated gene pool. In comparison with another vegetatively propagated outbreeder, *Vitis vinifera*, the average MAF of potato (0.14) is lower. *Vitis vinifera* has an average MAF of 0.24 [62]. In a population of 80 grapevine cultivars, over 80% of SNP variants had a MAF above 0.10, while in potato we found only 48% of the variants had a MAF above 0.10. We expect that wild species introgressions with low MAF contribute significantly to the high variant density in potato.

To investigate whether we could identify potential signatures of selection in the potato genome, we examined the nucleotide diversity of loci along the physical position of each chromosome. Nucleotide diversity values were very variable both between and within chromosomes. Chromosomes 5 and 11 exhibited highest nucleotide diversity. Introgression of resistance genes from wild species is a likely explanation, as chromosome 5 and 11 contain the largest clusters of resistance genes, conferring resistance to a wide variety of pathogens [63], [64]. In contrast, overall nucleotide diversity was reduced on chromosome 10. Some of the most conserved genes in the dataset are located near the position of the skin colour, tuber shape and eye depth QTL on chromosome 10 [65]–[67]. An eye-catching difference between commercial western germplasm and Latin American land races are the highly variable tuber shapes and anthocyanin pigmentation patterns in tuber skin and flesh. Hence this reduced nucleotide diversity could well reflect a selective sweep.

Within all chromosomes, individual genes were found with reduced nucleotide diversity as well. It is not possible at this stage to say whether these genes themselves, rather than closely linked loci are under selection, but some of these genes are good candidate genes for phenotypic traits under strong selection like day-length dependent tuberisation and some resistance traits. An example of a good direct candidate gene is the *CONSTANS* gene that has been shown to affect the day-length regulation of tuber induction in potato [68] and is the second most conserved gene we sampled on chromosome 2.

### Chloroplast Types and Population Structure

The presence of an extreme cytoplasmic bottleneck in cultivated potato has been described before [69]. Five main cpDNA types (A, S, C, W and T) and a number of sub-types have been described [70]. The A haplotype is most frequent in Group Andigena and the T haplotype in Chilean S. *tuberosum* and modern cultivars. Diploid *S. tuberosum* Group Phureja is assigned to the S-type and wild material like *S. vernei* and *S. demissum* to the W-type. Our phylogeny of chloroplast haplotype data supports previous work suggesting that most modern cultivars have chloroplasts resembling those of the *S. tuberosum* Group Tuberosum genepool (T-Type) [71], [72]. Two cultivars have a W-type haplotype originating from *S.vernei* (VE71–105 and VTN62-33-3) and thirteen cultivars have a W-type haplotype originating from *S. demissum* [73]. These chlorotypes have been introduced during introgression of resistance traits. More remarkably, four heirloom cultivars in our sample (cv. Belle De Fontenay, cv. Kepplestone Kidney, cv. Home Guard, and cv. Shamrock) have chloroplast haplotypes phylogenetically close to that from the *S. tuberosum* Group Phureja (S-Type). This chlorotype might represent the *S. tuberosum* Group Andigena type (A-Type) that was more common in cultivars from before the 1840s late blight (*Phytophtra infestance*) epidemic [74].

The genome-wide population structure of this study largely corresponds with the structure observed using AFLP markers [48]. While the AFLP study distinguished five divergent cultivar groups, we identified four, with one outlier forming its own group. Both analyses support genetic similarity within heirloom cultivars and within starch cultivars, but the high-throughput approach used here did not support separate groups of fresh consumption, processing (crisps or fries), and additional miscellaneous cultivars. Instead, PCA analysis suggested the existence of a cluster of cultivars originating from continental Europe, consisting mainly of processing cultivars, along with a cluster containing cultivars of mixed, world-wide origin. Given that the first three components of the principal component analysis account for less than 15% of the total variation, it seems that there is little population structure within the cultivated potato gene pool, as was also observed by D’hoop et al. (2010).

### Concordance between GBS and KASP Genotyping in an Autotetraploid

In diploids it is known that the accuracy of variant detection and genotyping depends on sequence depth and the mode of sexual reproduction (homozygous selfers vs. heterozygous outbreeders) [75], [76]. Genotyping variants in polyploid species, such as potato, is more challenging than in diploids, because a given gene may be represented not only by a number of different alleles, but also by different zygosity levels. We therefore tested the accuracy of genotype calls made by GBS by testing a small subset of binary SNPs using an independent KASP genotyping platform. The genotype calls of GBS were found to be over 99% consistent with KASP when scored as homozygous or heterozygous. We applied a genotype quality (GQ) parameter to account for differences in read depth requirement across all zygosity classes (nulliplex, simplex, duplex, triplex, and quadruplex). Over 95% of duplex calls (the zygosity class with lowest consistency between GBS and KASP) were consistent between the two genotyping methods at a threshold of GQ26. Overall concordance of the five zygosity classes was 98.4% at this threshold. In conclusion, for genotyping autotetraploids a GQ26 is recommended to achieve reliable genotype calls using our pipeline. In a more general sense, our results indicate that read depths of 60–80x are sufficient for reliable, dosage diagnostic genotype calls in GBS in autotetraploid potato.

### Association Analysis using GBS

The GBS dataset was tested for its performance in GWAS. Even though the data do not represent a complete genome, but a focussed survey at 807 loci randomly distributed across the genome, and although the number of 83 potato cultivars severely limits statistical power, several interesting traits could be mapped. It appeared that the 129,156 sequence variants provide a robust dataset to detect alleles influencing monogenic traits such as plant maturity and tuber flesh colour (this paper) and various other monogenic traits such as tuber shape and pathogen resistance (unpublished data). The sequence variants associated with tuber flesh colour and plant maturity are indeed located in and near the respective candidate genes *CHY2* [34] and *StCDF1*. The latter was recently shown to be the causal gene involved in potato day-length signalling [77]. We observed that a simplistic dominant model (0/1 data reflecting absence/presence of an allele) gave similar GWAS results as the more detailed additive model (0/1/2/3/4 copies of the allele). This makes sense, since 61% of the sequence variants have a MAF below 0.05. If such variants follow Hardy-Weinberg expectations, in practice the minor allele will be absent in ∼81% of the cultivars and simplex in ∼17% of the cultivars, while a duplex cultivar is hardly expected (1.4%). This notion, that many sequence variants have a low allele frequency, points to the advantage that the input GBS data does not require the more stringent genotype quality filtering necessary for accurate estimation of allele copy number.

GWAS requires marker densities surpassing the decay in LD. In this study only a fraction (2.1 Mb, 0.25%) of the 840 Mb potato genome was re-sequenced, but in most cases the genetic distance between our 807 candidate genes will be less than the estimated LD decay of 4–10 cM [48], [56]. For the data generated here, both short- and long-range LD still needs to be analysed. Although short-range LD could have been analysed, long-range LD requires the robust ordering of superscaffolds of the DM reference genome into physical chromosomes, preferentially in combination with an aligned high-resolution genetic linkage map. These maps and pseudo-molecules are currently being developed to estimate LD decay on both genetic (cM) and physical (bp) scale in the near future. We expect that the identified sequence variants and regions covered in this study are in LD with most functional alleles. We also expect that identification of haplotypes and phasing of the sequence variants can be achieved by using a ‘read-backed phasing’ approach (unpublished data) for use in a multiallelic GWAS analysis. Since a large number of genes in our study were either known candidate genes, or primary- and secondary metabolism genes, the annotated biochemical pathway may suggest a biological link between the gene underlying the sequence variants, and the QTL. Alternatively, the potato genome browser (http://solanaceae.plantbiology.msu.edu) will support the identification of candidate genes in proximity to QTL.

## Supporting Information

Supporting Information S1 **Supporting Tables and Figures.**
(DOC)

BED-file S1 **SureSelect baits mapped (MQ≥37) to the superscaffolds of the DM reference genome.**
(BED)

BED-file S2 **Accessible genome regions of the DM reference genome.**
(BED)

CSV-file S1 **Annotations for sequence variants identified in the accessible genome, including allele copy numbers of each of the 84 samples.**
(GZ) Click here for additional data file.

FASTA-file S1 **SureSelect RNA bait sequences.**
(FASTA)

FASTA-file S2 **SureSelect target sequences.**
(FASTA)

VCF-file S1 **Sequence variants and genotypes identified in the accessible potato DM genome of 84 samples.**
(GZ) Click here for additional data file.

VCF-file S2 **Sequence variants identified in the chloroplast genome.**
(VCF)

XLS-file S1 **Annotations for genomic SureSelect targets, including observed coverage in resequencing data.**
(XLS)

XLS-file S2 **Annotations for accessible genome regions, based on sequence data collected in this study.**
(XLS)

XLS-file S3 **Annotations of nucleotide diversity for accessible genes.**
(XLS)
