# Correction: A Next-Generation Sequencing Method for Genotyping-by-Sequencing of Highly Heterozygous Autotetraploid Potato

**DOI:** 10.1371/journal.pone.0141940

**Published:** 2015-10-28

**Authors:** Jan G. A. M. L. Uitdewilligen, Anne-Marie A. Wolters, Bjorn B. D’hoop, Theo J. A. Borm, Richard G. F. Visser, Herman J. van Eck

There is an error in the second sentence of the sub-section Nucleotide Diversity within the Results. The correct sentence is: Mean π of the covered genome was 1.07×10^−2^ (Table 4).
