# Supplementary material for: A Next-Generation Sequencing Method for Genotyping-by-Sequencing of Highly Heterozygous Autotetraploid Potato
Source: PLoS One. 2013 May 8;8(5):e62355. doi: 10.1371/journal.pone.0062355 (PMC3648547; doi:10.1371/journal.pone.0062355)
Supplement: Supporting Information S1 — Supporting Tables and Figures. (DOC) [file pone.0062355.s001.doc]

# Supporting Information

**Table A. Potato clones used in this experiment.**

| *Name^a^* | *Year of release* | *Origin^b^* | *Genotype Code^c^* | *Pool ID* | *Adapter Index ID* | *Mean read depth^d^* | *Bases with depth>15x (%)* | *Population cluster* | *Chloroplast type* |
| --- | --- | --- | --- | --- | --- | --- | --- | --- | --- |
| 1256A(23) = Black 1256 | ? | GB | P80001 | P1 | PEM01 | 25.60 | 65.0 | 1 | Type T(a) |
| Arrow | 2004 | HOL | P80031 | P1 | PEM02 | 30.28 | 71.7 | 3 | Type T(a) |
| Innovator | 1999 | HOL | P80106 | P1 | PEM03 | 56.94 | 89.5 | 3 | Type T(a) |
| Vitelotte Noir | <1815 | FRA | P80232 | P1 | PEM04 | 67.37 | 91.1 | 3 | Type T(b) |
| Ve 71-105 | 1971 | HOL | P80206 | P1 | PEM05 | 73.28 | 92.6 | 2 | Type Wv |
| Aurora | 1972 | HOL | P80035 | P1 | PEM06 | 15.41 | 48.4 | 3 | Type T(b) |
| Yam | <1787 | GB | P80221 | P1 | PEM07 | 43.79 | 80.3 | 1 | Type T(b) |
| Ultimus | 1935 | HOL | P80199 | P1 | PEM08 | 84.92 | 94.9 | 5 | Type T(b) |
| Shamrock | <1900 | IRL | P80182 | P1 | PEM09 | 41.75 | 79.8 | 1 | Type 4(a) |
| Princess | 1998 | BRD | P80234 | P1 | PEM10 | 65.25 | 89.5 | 5 | Type Wd(a) |
| Aveka | 2001 | HOL | P8B119 | P1 | PEM11 | 35.36 | 72.9 | 2 | Type T(a) |
| Eos | 2000 | HOL | P80076 | P1 | PEM12 | 36.50 | 77.3 | 3 | Type T(a) |
| Nomade | 1995 | HOL | P80151 | P2 | PEM01 | 80.72 | 90.3 | 2 | Type T(a) |
| Markies | 1997 | HOL | P80139 | P2 | PEM02 | 78.43 | 90.0 | 5 | Type T(a) |
| Adretta | 1975 | DDR | P80006 | P2 | PEM03 | 46.08 | 78.2 | 5 | Type Wd(c) |
| Kerpondy | 1949 | FRA | P80118 | P2 | PEM04 | 30.98 | 68.7 | 5 | Type T(a) |
| Victoria | 1997 | HOL | P80208 | P2 | PEM05 | 62.26 | 81.9 | 5 | Type T(a) |
| Biogold | 2004 | HOL | P80045 | P2 | PEM06 | 34.57 | 74.0 | 3 | Type T(a) |
| Pentland Dell | 1961 | GB | P80159 | P2 | PEM07 | 14.65 | 46.6 | 1 | Type T(a) |
| Toyoshiro | 1976 | JAP | P80192 | P2 | PEM08 | 43.22 | 73.1 | 3 | Type T(b) |
| Kepplestone Kidney | <1900 | GB | P80117 | P2 | PEM09 | 50.52 | 81.1 | 1 | Type 4(b) |
| Fontane | 1999 | HOL | P80088 | P2 | PEM10 | 67.81 | 83.7 | 5 | Type T(a) |
| Felsina | 1992 | HOL | P80082 | P2 | PEM11 | 74.91 | 84.7 | 5 | Type T(a) |
| Fianna | 1987 | HOL | P80084 | P2 | PEM12 | 64.53 | 85.8 | 3 | Type T(a) |
| Industrie | 1900 | GER | P80105 | P3 | PEM01 | 62.41 | 90.8 | 5 | Type T(a) |
| Samba | 1989 | FRA | P80176 | P3 | PEM02 | 60.89 | 87.4 | 3 | Type T(a) |
| Libertas | 1946 | HOL | P80131 | P3 | PEM03 | 96.41 | 98.2 | 3 | Type T(a) |
| Vk 69-491 | 1969 | HOL | P80211 | P3 | PEM04 | 92.02 | 96.5 | 2 | Type T(a) |
| Mpi 19268 | 1940 | GER | P80145 | P3 | PEM05 | 80.15 | 93.9 | 5 | Type Wd(a) |
| Agria | 1985 | BRD | P80008 | P3 | PEM06 | 97.72 | 97.2 | 5 | Type T(a) |
| Ajiba | 1992 | HOL | P80009 | P3 | PEM07 | 107.59 | 97.7 | 3 | Type T(a) |
| Y 66-13-636 | 1966 | HOL | P80220 | P3 | PEM08 | 127.26 | 97.8 | 3 | Type T(a) |
| Herald | 1928 | GB | P80099 | P3 | PEM09 | 49.47 | 84.8 | 3 | Type T(a) |
| Anosta | 1975 | HOL | P80022 | P3 | PEM10 | 145.05 | 99.1 | 3 | Type T(a) |
| Ve 74-45 | 1974 | HOL | P80207 | P3 | PEM11 | 99.76 | 96.2 | 2 | Type T(a) |
| Tasso | 1963 | BRD | P80188 | P3 | PEM12 | 84.95 | 95.5 | 3 | Type Wd(c) |
| Nicola | 1973 | BRD | P80147 | P4 | PEM01 | 116.71 | 98.0 | 5 | Type T(a) |
| Kuras | 1996 | HOL | P80122 | P4 | PEM02 | 163.07 | 99.3 | 2 | Type Wd(b) |
| Ehud | 1965 | HOL | P80073 | P4 | PEM03 | 98.06 | 98.1 | 3 | Type Wd(c) |
| Hindenburg | 1916 | GER | P80101 | P4 | PEM04 | 141.58 | 99.4 | 5 | Type Wd(ba) |
| Golden Wonder | 1906 | GB | P80095 | P4 | PEM05 | 104.55 | 98.2 | 1 | Type T(a) |
| Vtn 62-33-3 | 1962 | HOL | P80214 | P4 | PEM06 | 105.78 | 96.7 | 2 | Type Wv |
| Avenance | 2005 | HOL | P80037 | P4 | PEM07 | 94.48 | 97.1 | 2 | Type Wd(c) |
| Ve 70-9 | 1970 | HOL | P80205 | P4 | PEM08 | 137.6 | 99.1 | 2 | Type T(a) |
| Hansa | 1957 | BRD | P80098 | P4 | PEM09 | 106.01 | 97.1 | 5 | Type T(a) |
| Ackersegen | 1929 | GER | P80003 | P4 | PEM10 | 174.54 | 99.3 | 5 | Type T(a) |
| Umatilla Russet | 1998 | USA | P80200 | P4 | PEM11 | 177.2 | 99.1 | 3 | Type T(a) |
| Laura | 1998 | BRD | P80128 | P4 | PEM12 | 100.42 | 96.7 | 5 | Type T(a) |
| Picasso | 1994 | HOL | P80161 | P5 | PEM01 | 73.59 | 90.5 | 3 | Type T(a) |
| Obelix | 1988 | HOL | P80153 | P5 | PEM02 | 61.93 | 89.4 | 3 | Type T(a) |
| Monoploid 1-3 551 | NA | NA | NA | P5 | PEM03 | 51.72 | 81.8 | 4 | Type S |
| Mondial | 1987 | HOL | P80143 | P5 | PEM04 | 46.45 | 82.3 | 3 | Type T(a) |
| Exquisa | 1992 | BRD | P80080 | P5 | PEM05 | 47.76 | 81.4 | 5 | Type T(a) |
| Frieslander | 1990 | HOL | P80090 | P5 | PEM06 | 81.26 | 93.0 | 3 | Type T(a) |
| Ballydoon | 1931 | GB | P80038 | P5 | PEM07 | 29.91 | 70.0 | 1 | Type T(a) |
| Bintje | 1910 | HOL | P80044 | P5 | PEM08 | 90.97 | 94.3 | 3 | Type T(a) |
| Wisent | 2005 | HOL | P80219 | P5 | PEM09 | 60.17 | 88.9 | 2 | Type Wd(b) |
| Irish Queen | <1900 | GB | P80109 | P5 | PEM10 | 69.21 | 91.4 | 1 | Type T(a) |
| Festien | 2000 | HOL | P80083 | P5 | PEM11 | 47.83 | 80.5 | 2 | Type Wd(c) |
| Hermes | 1973 | AUT | P80100 | P5 | PEM12 | 90.71 | 93.6 | 5 | Type T(a) |
| Katahdin | 1932 | USA | P80115 | P6 | PEM01 | 73.63 | 93.5 | 3 | Type T(a) |
| Clivia | 1962 | BRD | P80053 | P6 | PEM02 | 52.1 | 84.7 | 5 | Type T(a) |
| Gladstone | 1932 | GB | P80093 | P6 | PEM03 | 33.7 | 72.6 | 1 | Type T(a) |
| Ditta | 1989 | AUT | P80062 | P6 | PEM04 | 92.33 | 96.3 | 5 | Type T(a) |
| Estima | 1973 | HOL | P80079 | P6 | PEM05 | 100.85 | 96.3 | 3 | Type T(a) |
| Arran Pilot | 1930 | GB | P80029 | P6 | PEM06 | 32.27 | 72.0 | 3 | Type T(a) |
| Tinwald's Perfection | 1914 | GB | P80191 | P6 | PEM07 | 65.09 | 90.2 | 1 | Type T(a) |
| Albion | 1895 | HOL | P80010 | P6 | PEM08 | 50.40 | 85.3 | 3 | Type T(a) |
| Amyla | 1999 | FRA | P80021 | P6 | PEM09 | 39.93 | 77.3 | 3 | Type Wd(a) |
| Alpha | 1925 | HOL | P80015 | P6 | PEM10 | 107.47 | 97.8 | 3 | Type T(a) |
| Winston | 1992 | GB | P80218 | P6 | PEM11 | 45.5 | 79.4 | 3 | Type T(a) |
| Great Scot | 1909 | GB | P80097 | P6 | PEM12 | 57.07 | 89.3 | 1 | Type T(a) |
| Civa | 1960 | HOL | P80052 | P7 | PEM01 | 24.71 | 65.7 | 5 | Type T(a) |
| Arran Chief | 1911 | GB | P80028 | P7 | PEM02 | 32.76 | 76.1 | 1 | Type T(a) |
| Usda 96-56 | ? | USA | P80203 | P7 | PEM03 | 47.5 | 82 | 3 | Type T(a) |
| Belle de Fontenay | 1885 | FRA | P80040 | P7 | PEM04 | 29.5 | 69.9 | 1 | Type 4(a) |
| Kartel | 1994 | HOL | P80114 | P7 | PEM05 | 45.37 | 83.5 | 2 | Type Wd(c) |
| Home Guard | 1943 | GB | P80102 | P7 | PEM06 | 36.29 | 73.4 | 1 | Type 4(a) |
| Daisy | 1998 | FRA | P80057 | P7 | PEM07 | 23.14 | 64.1 | 3 | Type T(b) |
| Voran | 1931 | GER | P80212 | P7 | PEM08 | 60.06 | 87 | 3 | Type T(a) |
| Cherie | 1997 | FRA | P80050 | P7 | PEM09 | 41.95 | 82.6 | 3 | Type T(a) |
| Mercator | 1999 | HOL | P80141 | P7 | PEM10 | 77.38 | 93.4 | 2 | Type Wd(c) |
| Vivaldi | 1998 | HOL | P80210 | P7 | PEM11 | 69.19 | 91.2 | 5 | Type T(a) |
| Charlotte | 1981 | FRA | P80049 | P7 | PEM12 | 32.81 | 75.5 | 5 | Type T(a) |

^a^ Name of cultivar, progenitor clone, or monoploid clone
^b^ Country of first market release
^c^ Same as P8 codes defined by D’hoop et al. (2008)
^d^ MQ13 of covered regions

**Table B. Sequences of the twelve custom-ordered indexed adapter pairs.** Nucleotides in bold are included to facilitate sticky-end ligation. The index sequence is underlined. Oligos were HPLC purified.

| *Adapter pair* | *Strand A (5’ –> 3’)* | *Strand B (5 ’–> 3’)* |
| --- | --- | --- |
| PEM01 | ACACTCTTTCCCTACACGACGCTCTTCCGATCTTGCA***T** | P-TGCAAGATCGGAAGAGCGGTTCAGCAGGAATGCCGAG |
| PEM02 | ACACTCTTTCCCTACACGACGCTCTTCCGATCTTCAG***T** | P-CTGAAGATCGGAAGAGCGGTTCAGCAGGAATGCCGAG |
| PEM03 | ACACTCTTTCCCTACACGACGCTCTTCCGATCTTACG***T** | P-CGTAAGATCGGAAGAGCGGTTCAGCAGGAATGCCGAG |
| PEM04 | ACACTCTTTCCCTACACGACGCTCTTCCGATCTGTGC***T** | P-GCACAGATCGGAAGAGCGGTTCAGCAGGAATGCCGAG |
| PEM05 | ACACTCTTTCCCTACACGACGCTCTTCCGATCTGGTC***T** | P-GACCAGATCGGAAGAGCGGTTCAGCAGGAATGCCGAG |
| PEM06 | ACACTCTTTCCCTACACGACGCTCTTCCGATCTGCGT***T** | P-ACGCAGATCGGAAGAGCGGTTCAGCAGGAATGCCGAG |
| PEM07 | ACACTCTTTCCCTACACGACGCTCTTCCGATCTCTGA***T** | P-TCAGAGATCGGAAGAGCGGTTCAGCAGGAATGCCGAG |
| PEM08 | ACACTCTTTCCCTACACGACGCTCTTCCGATCTCGTA***T** | P-TACGAGATCGGAAGAGCGGTTCAGCAGGAATGCCGAG |
| PEM09 | ACACTCTTTCCCTACACGACGCTCTTCCGATCTCATG***T** | P-CATGAGATCGGAAGAGCGGTTCAGCAGGAATGCCGAG |
| PEM10 | ACACTCTTTCCCTACACGACGCTCTTCCGATCTATAC***T** | P-GTATAGATCGGAAGAGCGGTTCAGCAGGAATGCCGAG |
| PEM11 | ACACTCTTTCCCTACACGACGCTCTTCCGATCTACAT***T** | P-ATGTAGATCGGAAGAGCGGTTCAGCAGGAATGCCGAG |
| PEM12 | ACACTCTTTCCCTACACGACGCTCTTCCGATCTAACT***T** | P-AGTTAGATCGGAAGAGCGGTTCAGCAGGAATGCCGAG |

* = phosphorothioate bond
P- = phosphate group

**Table C. Primers used for amplification of hybrid-selected DNA.**

| *Name* | *Sequence (5’ –> 3’)* |
| --- | --- |
| PCR_PE2.0 | CAAGCAGAAGACGGCATACGAGATCGGTCTCGGCATTCCTGCTGAACCGCTCTTCCGATC*T |
| PCR_PE1.0 | AATGATACGGCGACCACCGAGATCTACACTCTTTCCCTACACGACGCTCTTCCGATC*T |

* = phosphorothioate bond

**Table D. Primers for quantification of enrichment libraries.**

| *Name* | *Sequence (5’ –> 3’)* |
| --- | --- |
| qPCR_1.1 | AATGATACGGCGACCACCGAGAT |
| qPCR_2.1 | CAAGCAGAAGACGGCATACGA |

**Figure A. HiSeq2000 read-pairs.** Each graph represents the number of read-pairs obtained from twelve potato cultivars, loaded as a single pool, for a total of seven pools. Most pools required only one sequencing lane, but pools 2, 3, and 4 exhibited reverse-read sequencing failures and therefore required multiple sequencing lanes, as noted in their respective panels. Including repetitions a total of 11 HiSeq2000 lanes were sequenced. Green bars represent the number of read-pairs with valid indices in both reads. Yellow, orange and red bars represent the number of read-pairs where the forward, the reverse, or neither of the indices were valid. Coloured bars interrupted with a line indicate separate read-pairs obtained during the first attempt (lower part) or from the second or third attempts (middle or upper part of the stacked bar, respectively).

**Figure B.** Nucleotide diversity (*π × 10^-3^*) of sequenced contigs (average size 874±656 bp, mean±SD) across chromosomes ordered in accordance with current pseudomolecule order. Nucleotide diversity at coding, non-coding, and all regions are coloured in red, blue, and black, respectively. The dashed line represents the average genome wide nucleotide diversity (10.7×10^-3^).
